# Supplementary material for: Phosphatidic Acid Stimulates Lung Cancer Cell Migration through Interaction with the LPA1 Receptor and Subsequent Activation of MAP Kinases and STAT3
Source: Biomedicines. 2023 Jun 23;11(7):1804. doi: 10.3390/biomedicines11071804 (PMC10376810; doi:10.3390/biomedicines11071804)
Supplement: Supplementary file 1 [file biomedicines-11-01804-s001.zip › biomedicines-2456825-supplementary.pdf]

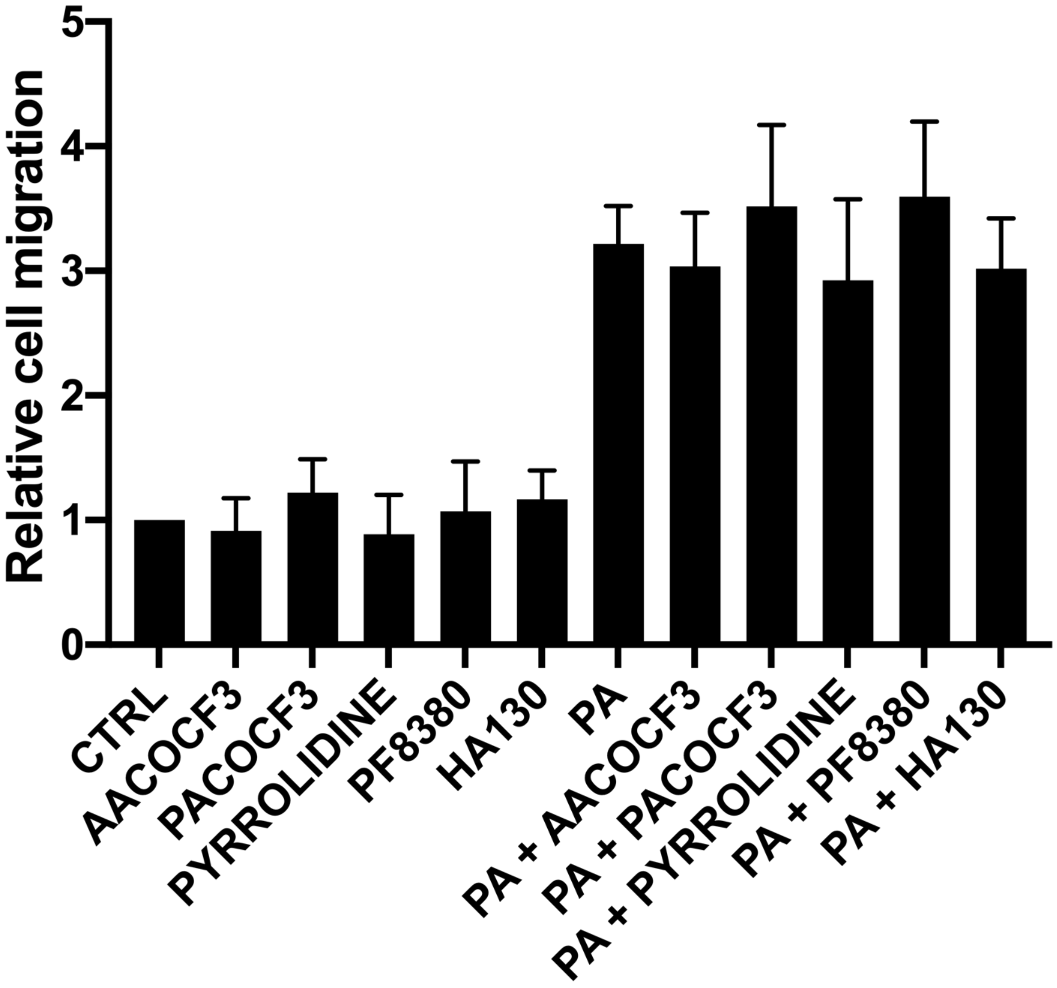

Supplementary Figure S1. Lack of effect of PLA2 and autotaxin inhibitors on PA-stimulated A549 lung cancer cell migration. Cells were preincubated for 90 min with or without the specific $cPLA_2$ inhibitors AACOCF3 (20 µM), PACOCF3 (20 µM) and pyrrolidine (1 µM), or with the autotaxin inhibitors PF8380 (300 nM) and HA130 (300 nM) as indicated, before stimulation with PA (10 µM) and cell migration was measured as detailed in Materials and Methods. Data are expressed relative to the control value and are given as the mean ± SD of 3 independent experiments performed in duplicate.
